# Tetrabenazine-Responsive Choreo-Ballism and Akathisia After Cardioversion and Coronary Angiography: A Case Report

**DOI:** 10.7759/cureus.106503

**Published:** 2026-04-06

**Authors:** Mostafa Fatheel, Rami Karkout

**Affiliations:** 1 Clinical Department of Neurology, Region Östergotland, Linköping, SWE

**Keywords:** chorea, coronary artery angiography, electrical cardioversion, movement disorders, vmat2 inhibitor

## Abstract

Hyperkinetic movement disorders after routine cardiac procedures are rare yet disabling and often misdiagnosed. We report the case of a 76-year-old man who developed abrupt, generalized choreo-ballism with severe akathisia one day after coronary angiography. Metabolic and inflammatory serum studies were unremarkable, brain magnetic resonance imaging (MRI) demonstrated no acute causative lesion, including no basal ganglia abnormality, and cerebrospinal fluid (CSF) analysis was non-inflammatory with negative infectious, autoimmune, and paraneoplastic testing. Low-dose tetrabenazine produced rapid (>80%) and sustained remission, permitting drug discontinuation after 16 months without relapse. When choreo-ballism emerges after cardiac interventions, transient basal ganglia dysfunction related to peri-procedural hypoxia or contrast neurotoxicity may be considered as a possible mechanism, and early vesicular monoamine transporter type 2 (VMAT-2) inhibition may facilitate recovery.

## Introduction

Subacute choreo-ballism and severe akathisia after routine cardiac interventions appear to be exceptionally rare. In the available literature, contrast-induced encephalopathy after diagnostic coronary angiography has been estimated at 0.05-0.11% and after percutaneous coronary intervention (PCI) at 0.3-0.4% [1], whereas post-hypoxic movement disorders have been reported in 26.4% of adults after cardiac arrest [2]; however, generalized choreo-ballism with akathisia in this setting remains distinctly uncommon. We describe an elderly patient who developed violent, generalized hyperkinesia and inner restlessness shortly after electrical cardioversion and diagnostic coronary angiography procedures not typically associated with movement disorders. By detailing the clinical course, investigative work-up, and striking response to low-dose tetrabenazine, this report underscores the vulnerability of basal ganglia circuitry to transient peri-procedural hypoxia or contrast-related dysfunction and highlights the importance of early recognition and tailored vesicular monoamine transporter type 2 (VMAT-2) inhibitor therapy for optimal recovery.

## Case presentation

**Figure 1:**
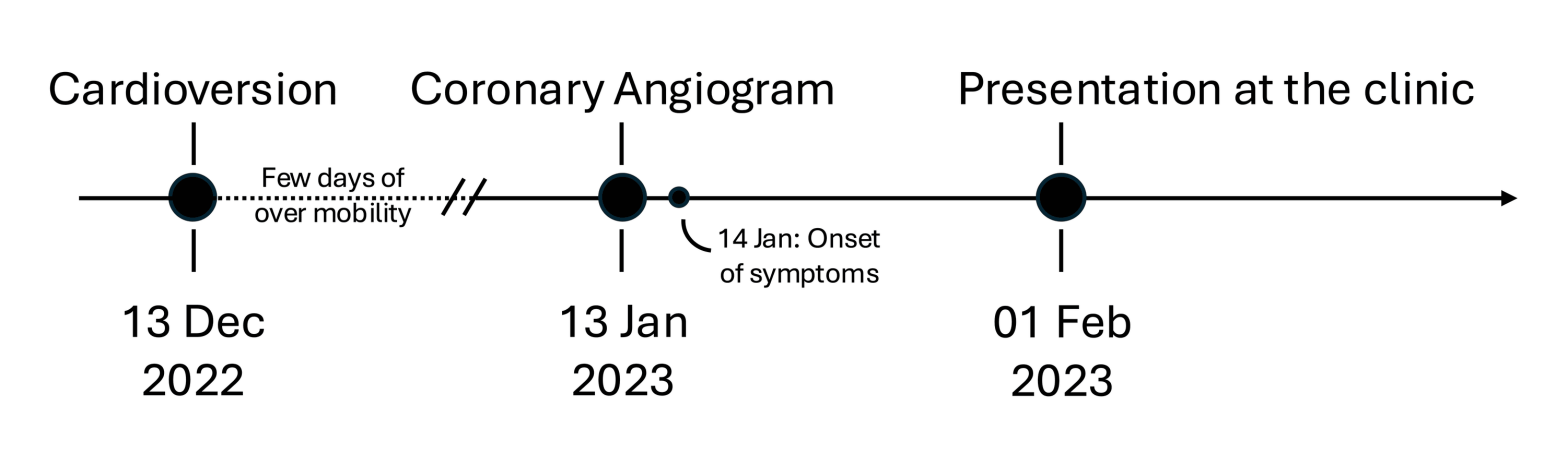
Timeline of cardiac procedures, symptom onset, and presentation at the clinic Chronological overview of the patient's clinical course, showing electrical cardioversion on 13 December 2022 with a brief transient hyperkinetic episode, coronary angiography on 13 January 2023, and abrupt onset of persistent generalized choreo-ballism and akathisia on 14 January 2023.

A 76-year-old man, still working as a bus driver, presented to the emergency department on 1 February 2023 with an 18-day history of violent, involuntary limb movements and an intense inner restlessness that prevented him from sitting, standing, or sleeping. The symptoms had begun abruptly on 14 January 2023, one day after a diagnostic coronary angiogram. Notably, he had experienced a fleeting, much milder episode of "over-mobility" in all four limbs immediately after electrical cardioversion for new-onset atrial fibrillation on 13 December 2022 (Figure 1).

Diagnostic coronary angiography with pressure measurements was performed via the right radial artery using a 6F Terumo introducer (Tokyo, Japan). At the time of vascular access, the patient's oxygen saturation was 93%, his heart rate was 60 bpm, and his blood pressure was 155/71 mmHg. The patient did not require any oxygen supplementation during the procedure. The procedure utilized a JL 3.5 guiding catheter and a PressureWire X (Abbott, Lake County, Illinois, United States) to measure fractional flow reserve (FFR). The procedure revealed a calcified left main coronary artery and the first obtuse marginal (OM1) branch, both of which were assessed with FFR and found to have no hemodynamic significance. A chronic total occlusion (CTO) was identified in the right coronary artery. Overall, the left coronary system showed no significant stenoses, though there was considerable calcification. Vascular closure was achieved using a TR-band, which was applied at 09:15 and inflated with 16 mL of air. A StatSeal device (Biolife, Sarasota, Florida, United States) was also applied and is to remain in place for 24 hours. Based on the clinical presentation and angiographic findings, conservative management is recommended regarding the CTO in the right coronary artery.

The earlier transient episode followed electrical cardioversion for new-onset atrial fibrillation. The procedure was performed under anesthesia with 120 mg of propofol and required a single 150 joule shock. At that time, the phenomenon had resolved within days. Besides hypertension, atrial fibrillation (treated with apixaban 5 mg twice daily since November 2022), and hyperlipidemia (rosuvastatin 20 mg nightly), he had no medical or family history of neurological disease and had never taken dopamine-blocking agents.

General/mental status and cranial nerves

On examination, he appeared exhausted but alert and fully oriented. Speech was mildly dysarthric. Pupils were equal and reactive; cranial nerve testing, including extra-ocular movements, visual fields, facial strength, palate elevation, and tongue protrusion, was unremarkable.

Motor and coordination findings

Motor assessment showed normal bulk, full strength, and normal tone without rigidity or spasticity. Deep tendon reflexes were brisk in the biceps, triceps, brachioradialis, and patellae; Achilles reflexes were appropriate and plantar responses flexor. Finger-nose testing disclosed mild bilateral dysmetria, and rapid alternating movements were slowed.

Phenomenology

Throughout the examination, he exhibited frequent multifocal involuntary movements involving the arms, legs, neck, and jaw. These consisted predominantly of irregular, flowing choreiform movements with intermittent larger amplitude, ballistic flinging components. In addition, brief superimposed shock-like jerks were observed. The movements were approximately 20/min and reportedly persisted during sleep. In addition, the patient described pronounced inner restlessness with a persistent urge to move, resulting in an inability to sit or stand still and severe sleep disturbance; this history was corroborated by his accompanying wife. These symptoms were considered suggestive of akathisia (see Video 1).

**Video 1:** Subacute generalized hyperkinesia with akathisia after coronary angiography (acute presentation)

**Figure 2:**
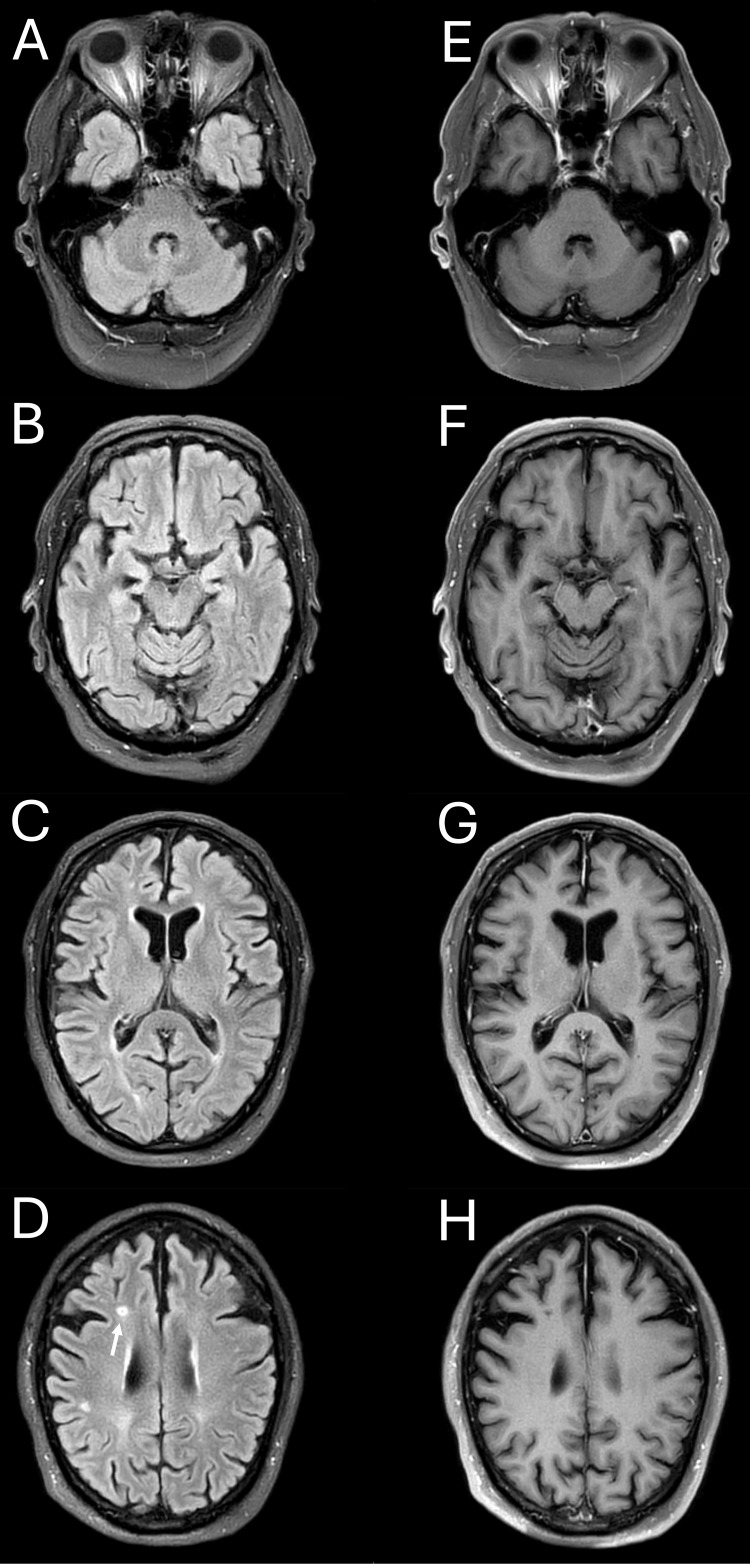
Brain MRI (11 February 2023), axial FLAIR, and T1-weighted images pre- and post-gadolinium (A-D) Pre-contrast FLAIR and (E-H) post-contrast T1-weighted axial images (gadolinium). MRI demonstrates a small right frontal subcortical lacunar infarct (arrow) and scattered supratentorial white matter hyperintensities. No diffusion restriction, oedema, basal ganglia signal abnormality, or contrast enhancement is seen. MRI: magnetic resonance imaging; FLAIR: fluid-attenuated inversion recovery

Initial laboratory studies, including serum electrolytes, renal and liver panels, glucose, thyroid function, vitamin B₁₂, folate, HbA1c, magnesium, parathyroid hormone, inflammatory markers, antinuclear and antineutrophil cytoplasmic antibodies, and viral serologies, were normal, apart from a weakly positive homogeneous ANA titre. Brain magnetic resonance imaging (MRI) with and without gadolinium (11 February 2023) demonstrated a small right subcortical frontal lacunar infarct and scattered supratentorial white matter hyperintensities but no diffusion restriction, oedema, basal ganglia signal change, or contrast enhancement (Figure 2). The infarct was considered unlikely to be causative, as neither its anatomical location nor its temporal profile on imaging was consistent with the abrupt onset of motor symptoms.

Cerebrospinal fluid (CSF) obtained on 3 February showed no cells, normal protein, glucose, and lactate, negative oligoclonal bands, and a mildly elevated total tau (528 ng/L; reference <409 ng/L); infectious, autoimmune, and paraneoplastic antibody panels were negative. Thoracic computed tomography (CT) and whole-body screening revealed no malignancy.

Empirical clonazepam 0.5 mg three times daily provided only partial symptomatic relief. Quetiapine up-titrated to 25 mg three times daily lessened agitation but not the hyperkinesia. Because of the temporal relationship to apixaban, the anticoagulant was paused on 6 March, switched briefly to rivaroxaban, and ultimately discontinued; no immediate neurological change ensued. Cardioversion was repeated uneventfully on 6 April.

On 25 April 2023, tetrabenazine (Tetmodis) was introduced at 12.5 mg three times daily. The initial tetrabenazine dose was chosen in accordance with local treatment guidelines, which recommend starting at 12.5 mg once to three times daily and then titrating according to clinical response and tolerability. Within one month, the frequency and amplitude of involuntary movements fell markedly, nocturnal sleep normalized, and the patient regained independent ambulation and self-care.

Serial reviews documented steady improvement. By 2 June 2023, the hyperkinesia had virtually disappeared (see Video 2).

**Video 2:** Near-complete resolution of generalized choreo-ballism and akathisia after tetrabenazine

Only mild dysarthria and subtle personality changes (increased emotional lability and drive) persisted. Tetrabenazine was tapered gradually and stopped in August 2024, after which the patient experienced a brief period of irritability without recurrence of abnormal movements. At his final evaluation in October 2024, he was asymptomatic, neurologically intact, and off all antidyskinetic medication and had resumed part-time work as a bus driver. The clinical course, therefore, represented a subacute, generalized choreo-ballism with akathisia that remitted completely over 15 months, most likely facilitated by low-dose tetrabenazine.

## Discussion

The present case highlights an unusual constellation of subacute, generalized choreo-ballism accompanied by severe akathisia arising days after electrical cardioversion and coronary angiography. Although hyperkinetic phenomena have been described after global anoxic events, they remain rare in the post-cardiac procedure setting and can easily be mistaken for agitation or anxiety.

Potential pathophysiological mechanisms

A plausible mechanism is transient basal ganglia dysfunction related to peri-procedural hypoxic-ischaemic stress. In a multicentre series of adults resuscitated from cardiac arrest, up to 26.4% developed new movement disorders, most commonly myoclonus, chorea, or dystonia, with MRI often demonstrating basal ganglia injury rather than cortical laminar necrosis [2]. Experimental and clinical studies likewise suggest that the striatum is particularly vulnerable to even mild energy deprivation [3,4].

Coronary angiography also raises the possibility of contrast-related neurotoxicity. Iodinated contrast can disrupt the blood-brain barrier and cause contrast-induced encephalopathy [5]. A recent review of 127 contrast-induced encephalopathy cases found that 28% of patients manifested motor phenomena [6], although symptoms typically appear within hours and resolve in <48 hours [1], unlike the persistent course in our patient. Procedure-related microembolism was also considered, but MRI showed only a small right frontal subcortical lacunar infarct, which was anatomically remote from basal ganglia motor circuits and not temporally consistent with the abrupt onset of symptoms [7].

Differential diagnostic considerations

Alternative causes of chorea were systematically evaluated. Non-ketotic hyperglycaemia can produce reversible metabolic chorea, typically with characteristic T1 hyperintensity in the striatum and improvement after glycaemic correction [8,9], features not present in this case. Autoimmune and paraneoplastic chorea were also considered, but serum and CSF antibody studies were negative, and no malignancy emerged during follow-up [10].

Drug-related causes were reviewed as well. Although direct oral anticoagulants have occasionally been associated with encephalopathy or disequilibrium [11], isolated choreiform syndromes appear exceptional, and the persistence of symptoms despite anticoagulant change argued against a primary drug effect.

Treatment response and clinical implications

Management required a dual focus: addressing the putative inciting factors and controlling the disabling movements. For symptom control, we adopted a step-wise pharmacological approach. Clonazepam provided modest relief but excessive sedation; low-dose quetiapine calmed the internal restlessness yet had little effect on chorea. VMAT-2 inhibition with tetrabenazine at 12.5 mg three times daily produced a rapid >80% reduction in jerk frequency, mirroring modern systematic review data showing the consistent efficacy of tetrabenazine (and its derivatives) across hyperkinetic disorders, with acceptable tolerability in low doses [12]. We maintained vigilant mood monitoring because tetrabenazine can unmask depression or worsen akathisia [13,14].

Overall, this case supports the hypothesis that transient functional disturbance of basal ganglia networks may occur after cardiac procedures even in the absence of a clear structural lesion on MRI [15,16]. Early recognition of this possibility, together with exclusion of metabolic, immune, and structural mimics, may help guide timely symptomatic treatment. VMAT-2 inhibition may be a useful therapeutic option in selected cases, although causal inference from a single case remains limited [17].

## Conclusions

Subacute generalized choreo-ballism with akathisia may occur after otherwise uneventful cardiac interventions. In this case, the syndrome was considered most consistent with transient but functionally significant basal ganglia dysfunction, possibly related to peri-procedural hypoxic-ischaemic stress or, less likely, contrast neurotoxicity. Early recognition, exclusion of metabolic or immune mimics, and judicious use of VMAT-2 inhibitors, while monitoring for drug-induced akathisia, can yield excellent outcomes. Awareness of this entity among neurologists, cardiologists, and intensivists may contribute to good clinical outcomes.

## References

[1] Spina R, Simon N, Markus R, Muller DW, Kathir K (2017). Contrast-induced encephalopathy following cardiac catheterization. Catheter Cardiovasc Interv.

[2] Scheibe F, Neumann WJ, Lange C (2020). Movement disorders after hypoxic brain injury following cardiac arrest in adults. Eur J Neurol.

[3] Fugate JE (2017). Anoxic-ischemic brain injury. Neurol Clin.

[4] Waraich M, Mawdsley E (2024). Hypoxic ischaemic brain injury. Anaesth Intensive Care Med.

[5] Babalova L, Ruzinak R, Ballova J (2021). Contrast-induced encephalopathy. Bratisl Lek Listy.

[6] Zhang Y, Zhang J, Yuan S, Shu H (2022). Contrast-induced encephalopathy and permanent neurological deficit following cerebral angiography: a case report and review of the literature. Front Cell Neurosci.

[7] Ito H, Uchida M, Sase T (2018). Risk factors of contralateral microembolic infarctions related to carotid artery stenting. Neurol Med Chir (Tokyo).

[8] Arecco A, Ottaviani S, Boschetti M, Renzetti P, Marinelli L (2024). Diabetic striatopathy: an updated overview of current knowledge and future perspectives. J Endocrinol Invest.

[9] Costa Hoffmeister M, Bonavides PS, Maurer Wiercinski V (2024). Hyperglycemia-induced hemichorea-hemiballismus syndrome - a systematic review. Arch Endocrinol Metab.

[10] Kyle K, Bordelon Y, Venna N, Linnoila J (2022). Autoimmune and paraneoplastic chorea: a review of the literature. Front Neurol.

[11] Rodrigues Cernadas E, Dionisio C, Estevão D, Vicente L (2021). Reversible neurological adverse reaction to apixaban. Eur J Case Rep Intern Med.

[12] Rosenthal LS, Farag M, Aziz NA, Bang J (2025). Vesicular monoamine transport inhibitors: current uses and future directions. Lancet.

[13] Vasireddy RP, Sokola B, Guduru Z (2020). New generation VMAT2 inhibitors induced parkinsonism. Clin Park Relat Disord.

[14] Vanegas-Arroyave N, Caroff SN, Citrome L (2024). An evidence-based update on anticholinergic use for drug-induced movement disorders. CNS Drugs.

[15] Niu N, Cui R (2017). Glucose hypermetabolism in contralateral basal ganglia demonstrated by serial FDG PET/CT scans in a patient with SLE chorea. Clin Nucl Med.

[16] Dambrain A, Boursot C, Cohen Tannugi K, Reichart J, Lacoeuille F (2024). Case report: utility of brain [18F]FDG PET/CT in the diagnosis of Sydenham's chorea. Front Nucl Med.

[17] Jankovic J (2016). Dopamine depleters in the treatment of hyperkinetic movement disorders. Expert Opin Pharmacother.

